# Genetic and Clinical Profiles of Pheochromocytoma and Paraganglioma: A Single Center Study

**DOI:** 10.3389/fendo.2020.574662

**Published:** 2020-12-11

**Authors:** Xiaosen Ma, Ming Li, Anli Tong, Fen Wang, Yunying Cui, Xuebin Zhang, Yushi Zhang, Shi Chen, Yuxiu Li

**Affiliations:** ^1^Department of Endocrinology, Key Laboratory of Endocrinology, National Health Commission of the People’s Republic of China, Peking Union Medical College Hospital, Peking Union Medical College, Chinese Academy of Medical Sciences, Beijing, China; ^2^Department of Clinical Laboratory, Peking Union Medical College Hospital, Peking Union Medical College, Chinese Academy of Medical Sciences, Beijing, China; ^3^Department of Endocrinology, Tongji Hospital, Tongji Medical College, Huazhong University of Science and Technology, Wuhan, China; ^4^Department of Urology, Peking Union Medical College Hospital, Peking Union Medical College, Chinese Academy of Medical Sciences, Beijing, China

**Keywords:** pheochromocytoma, paraganglioma, Chinese, genetics, genotype-phenotype relation

## Abstract

Pheochromocytoma/paraganglioma (PPGL) has a high genetic heterogeneity with 40% germline variants in known pathogenic genes. Data in Chinese on this aspect are scanty. To detect the genetic and clinical profile of Chinese PPGL patients, we examined the variants of 12 known germline pathogenic genes (*SDHA*, *SDHB*, *SDHC*, *SDHD*, *SDHAF2*, *FH*, *VHL*, *RET*, *NF1*, *MAX*, *TMEM127*, and *KIF1B*) by next-generation sequencing and Sanger sequencing in 314 Chinese PPGL subjects. Twenty nine percent of Chinese PPGL patients had germline variants and *SDHB* was the most frequently mutated (14.6%). The most frequent *SDHB* variants were in exon 2, exon 7, and IVS 7. Pathogenic variants were more likely to occur in metastatic PPGL patients, paragangliomas, and patients under 30, with the ratio being 50.7% (35/69), 35.9% (56/156), and 49.5% (52/105), respectively. Our cohort included 314 patients from a single setting. The genetic and clinical features of Chinese PPGL patients were unique in some aspects compared to their non-Chinese counterparts. Identification of genotype-phenotype relation can serve as an effective tool for genetic prioritization and clinical decision-making.

## Introduction

Pheochromocytoma (PCC) and paraganglioma (PGL) are tumors that originate from adrenal medulla, sympathetic ganglia and parasympathetic ganglia. With the development of the next-generation sequencing (1), about 40% of PCC and PGL (PPGL) bear a relationship with the germline variants of known pathogenic genes and the germline variants are genetically highly heterogeneous (2, 3). Over the last two decades, more than 20 genes have been found to be associated with the development of hereditary PPGL. Of them, *VHL*, *SDHB*, *SDHD*, *SDHA*, and *RET* are the most common pathogenic genes, while several novel pathogenic genes mutated with extremely low frequency, which were only found in one or several families (4–7). At present, the variants have been profiled in different races. In 2006, a review from the European Network for the Study of Adrenal Tumors (ENS@T) summarized germline variants (*VHL*, *SDHB*, *SDHD*, *RET*, and *NF1*) in 166 (25.9%) of 642 PPGL patients and *VHL* was reportedly the most frequently mutated gene (56/642, 8.7%) (8). Recently, a research conducted in Saudi Arabia exhibited that the variant rate was 36.6% in PPGL subjects and *SDHB* was the most common variant (9).Collectively, these studies showed that the variant rate and underlying genetic profile, to some extent, varied with different ethnic populations. Germline profiling of Chinese PPGLs from two centers found that the variant rate was 21.7% (55/261) and *VHL* was the most frequently mutated gene (10). To know the profile of germline variants and genotype-phenotype correlation in Chinese PPGLs from a single center, we examined the variants in 12 known germline pathogenic genes (*SDHA*, *SDHB*, *SDHC*, *SDHD*, *SDHAF2*, *FH*, *VHL*, *RET*, *NF1*, *MAX*, *TMEM127*, and *KIF1B*) in 314 Chinese PPGLs from a single center. Our study shows that twenty nine percent of Chinese PPGLs had germline variants and *SDHB* was the most frequently mutated (14.6%). We also found the mutation hotspots are different with Chinese and Western populations.

## Materials and Methods

### Patients and Sample Collection

Our study included 314 PPGL patients from the Peking Union Medical College Hospital, Beijing, China. Most patients had undergone surgery and were histopathologically diagnosed with PPGL. For the patients with unresectable or metastatic tumor, PPGL diagnosis was established on the basis of clinical features (headache, palpitations and sweating), increased 24-h urinary catecholamine excretion or plasma metanephrines and imaging tests. 99mTc-hydrazinonicotinyl-Tyr3-0ctreotide (99mTc-HYNIC-TOC) scintigraphy, Iodine-131 metaiodobenzylguanidine scintigraphy and contrast-enhanced thoracoabdominal-pelvic computed tomography (CT) were performed for almost all PPGLs. Some patients underwent head and neck enhanced CT or head and neck enhanced MRI examination. 18F-FDG-PET/CT or 68Ga-DOATATATE-PET/CT were performed to patients suspected of metastasis or PPGLs with multiple lesions. Blood samples of the patients were collected, with informed consents obtained from the patients and approval from the medical ethics committee of the hospital.

### Genomic DNA Preparation and Sanger Sequencing

Genomic DNA was extracted from the peripheral blood of PPGL patients by using blood DNA Midi Kit (Omega Bio-Tek, Norcross, Georgia, USA). For patients with metastatic PPGL, all the coding sequences and the intro-exon junctions of *SDHB* (NM_003000.3) were amplified and sequenced on an ABI3730 DNA analyzer (Applied Biosystems, CA, USA). For patients with head or neck PPGL, all the coding sequences and the intron-exon junctions of *SDHB* and *SDHD* (NM_003002.4) were amplified and sequenced. As for patients with clinically-diagnosed hereditary syndromes, such as multiple endocrine neoplasia Type 2 (MEN2) and Von Hippel-Lindau (VHL) disease, hot spots (10, 11, 16 exons) of *RET* (NM_020630.4) and all exons, including intro-exon junctions of *VHL* (NM_000551.3), were amplified and sequenced, respectively. The primers used for the PCR amplification were listed in **Supplementary Table 1**.

### Next-Generation Sequencing

For all the patients except those whose variants were detected by the aforementioned Sanger sequencing, next-generation sequencing covering *SDHA* (NM_004168.4), *SDHB* (NM_003000.3), *SDHC* (NM_003001.5), *SDHD* (NM_003002.4), *SDHAF2* (NM_017841.4), *VHL* (NM_000551.3), *RET* (NM_020630.4), *MAX* (NM_002382.5), *TEMEM127* (NM_017849.4), *FH* (NM_000143.4), *NF1* (NM_001042492.3), and *KIF1B* (NM_015074.3) was performed to examine the potential pathogenic germline variations.

The probes of target regions for relevant genes were designed against Agilent available online (http://www.agilent.com). Agilent SureSelectXT custom kit (Agilent Technologies, Palo Alto, CA) was used to generate sequence library according to instructions. Briefly, fragments of 180-280bp were produced by using a hydrodynamic shearing system (Covaris, Massachusetts, USA). The DNA fragments were end-repaired, A-tailed and adapter-ligated for Illumina sequencing. Then, size selection, PCR amplification and library hybridization were performed. Each captured library with an index was loaded onto the Illumina HiSeq X platform (Illumina Inc., San Diego, CA), and 150-bp paired-end reads were generated.

The fastq files were subjected to quality control to exclude unwanted sequences, including adapter-contaminated or low quality or unrecognizable nucleotides. The fastq files were aligned against the Human Reference Genome (UCSC hg19) by using the Burrows-Wheeler Aligner (BWA). Then, the SAM tools (11) were used to generate the final BAM file (12) to calculate the sequence coverage and depth by sorting the BAM files and performing repeated marking, partial realignment and base quality recalibration. At last, the Variant Call Format files obtained in the previous step were annotated by ANNOVAR (13).

Annotated variants with coverage over 10×, mutant allele frequency (MAF) < 0.01 in the 1,000 Genomes databases and in the exonic or splicing region (10 bp upstream and downstream of splicing sites) were retained (14). The synonymous variants were excluded. Non-synonymous SNVs were retained if at least two of the functional predictions by PolyPhen-2, SIFT, MutationTaster and CADD showed the SNV was damaging. “Benign” or “likely benign” variants in ClinVar were eliminated (15–18). American College of Genomic Medicine (ACMG) guidelines were used for the classification of variant pathogenicity (19). All the variants were confirmed by Sanger sequencing.

### SDHB Immunohistochemistry

Immunohistochemical procedures of SDHB (ZM-0162, Beijing Zhongshan Golden Bridge Biotechnology Co., Ltd., Beijing, China) were performed on formalin-fixed, paraffin-embedded (FFPE) tissue sections of PPGL according to the manufacturer’s recommendations (1:50 dilution). Positive SDHB staining was defined as strong granular staining in cytoplasm; negative SDHB as cytoplasm showing no staining with positive staining for the internal control.

### Multiplex Ligation Dependent Probe Amplification

Screening for large deletions of *SDHx* was carried out using the P226‐D1 Multiplex Ligation dependent Probe Amplification (MLPA) kit, following the manufacturer’s protocol (MRC‐Holland, Amsterdam, Netherlands). This P226‐D1 probemix contains probes for all exons of the SDHB, SDHC, SDHD, SDHAF2, and SDHAF1 genes. In addition, 13 reference probes are included in this probemix, detecting several different autosomal chromosomal locations.

Large deletions of VHL was detected using MLPA kit (P016-C2 VHL obtained from MRC‐Holland, Netherlands), following the protocols described by the manufacturer. This P016-C2 probemix contains 9 probes for VHL, 6 probes for genes located close to VHL, 2 probes on 3p which are further telomeric or centromeric from VHL, and 12 reference probes detecting sequences on other chromosomes.

Final products were separated using ABI 3730xl capillary electrophoresis (Life technologies, CA USA) and the electropherogram was evaluated using Coffalyser.Net.

### Statistical Analysis

Continuous variables with non-normal distribution were expressed as median (25%, 75%). Two independent samples were compared by using Mann-Whitney test. Categorical variables were presented as frequency counts and percentages. Association between two categorical variables was determined by Chi-square test. Kaplan-Meier survival analysis was employed as appropriate. A P < 0.05 was considered to be statistically significant. SPSS version 23.0 for Windows was used for all statistical analysis. All unverified VUS were not included in the genotype-phenotypic analysis.

## Results

### Patient Characteristics

**Figure 1:**
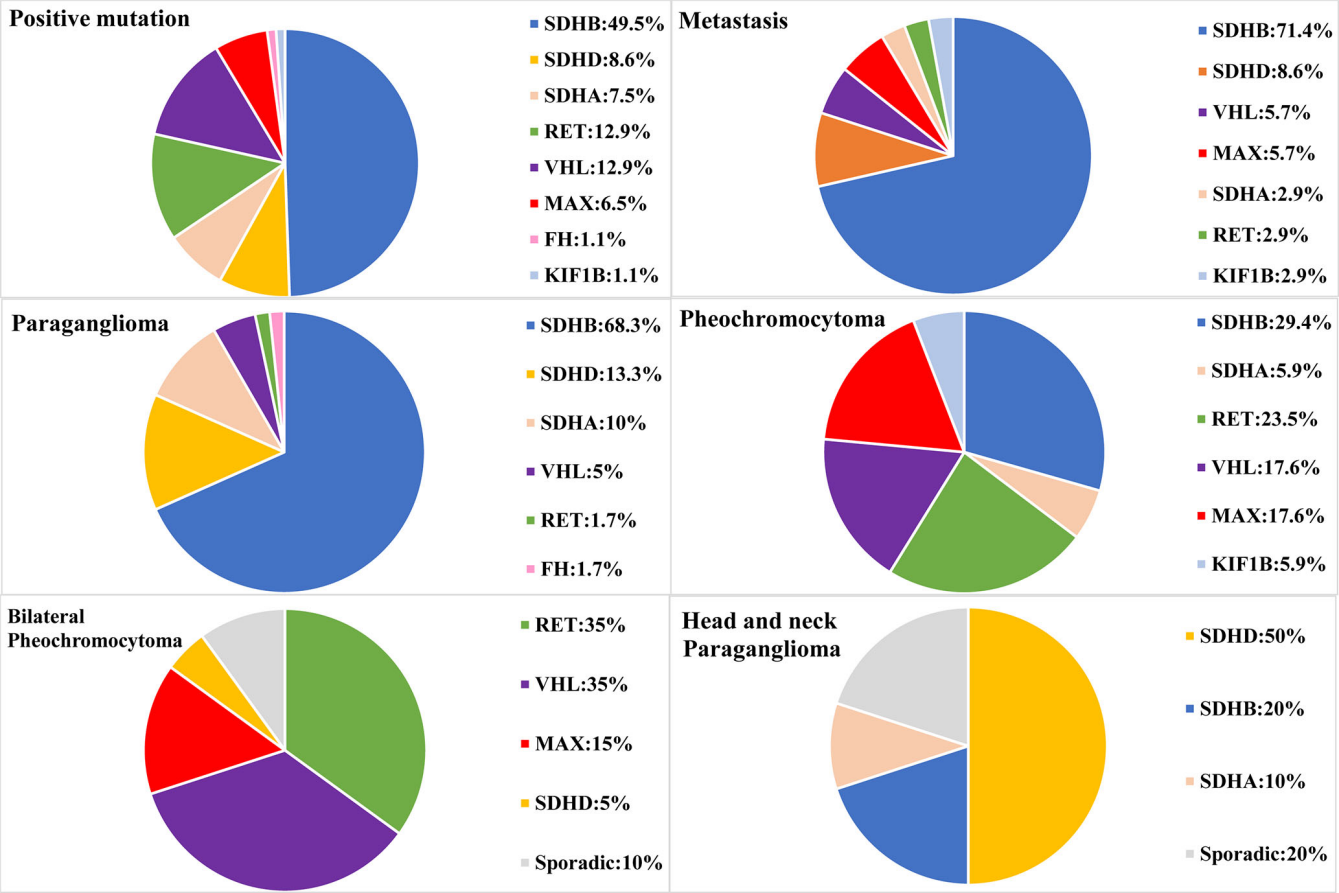
Genetic characteristics of PPGL patients with different phenotypes. Circular statistical graphic was used to illustrate the proportion of mutated gene in different clinical characteristic of PPGL.

Our study included 314 PPGL patients (165 females and 149 males), with a median age of 38 at diagnosis, ranging from 5 to 68. The clinical and genetic features of the patients are shown in **Figure 1** and **Tables 1** and **2**. In all, 314 patients, 69 (22.0%) had metastatic diseases. In addition, 156 (49.7%) patients had PGL in head and neck, thoracic, retroperitoneal, and pelvic locations; and 158 (50.3%) had PCC which contained 20 bilateral PCC; and 17 patients had tumors in both adrenal and extra-adrenal glands.

**Table 1:** Clinical and genetic characteristics of variant-positive patients.

No.	Gene	Exon	DNAmutation	Proteinchange	ACMG	AdjustedACMG	Novel/PMID/RS/	Gender	Age on set (year)	Age at dia. (year)	Delay(year)	Duration(month)	Metastasis	Age at meta.(y)	TumorLocation	TumorSize (cm)	FamilyHistory
1	*SDHB*	E2	c.79C>T	p.R27X	P	P	12000816	F	15	15	36	8	Yes	37	R- PGL	6	No
2	*SDHB*	E2	c.79C>T	p.R27X	P	P	12000816	M	36	37	1	12	Yes	37	R- PGL,P- PGL		No
3	*SDHB*	E2	c.137G>A	p.R46Q	P	P	12364472	F	37	41	4	48	No		R- PGL	15	No
4	*SDHB*	E2	c.137G>A	p.R46Q	P	P	12364472	M	10	10	4	–	Yes	14	R- PGL		No
5	*SDHB*	E2	c.136C>T	p.R46X	P	P	12618761	F	16	16	1		Yes	17	R- PGL		No
6	*SDHB*	E2	c.136C>T	p.R46X	P	P	12618761	F	25	31	0	72	Yes	30	P- PGL	8	No
7	*SDHB*	E2	c.136C>T	p.R46X	P	P	12618761	F	17	18	9	12	No		R- PGL	4	No
8	*SDHB*	E2	c.136C>T	p.R46X	P	P	12618761	F	20	21	0	5	Yes	21	R- PGL		No
9	*SDHB*	E2	c.136C>T	p.R46X	P	P	12618761	M	18	18	21	6	Yes	34	PCC	20	No
10	*SDHB*	E2	c.136C>T	p.R46X	P	P	12618761	F	25	26	6	12	Yes	29	R- PGL	4.7	No
11	*SDHB*	E2	c.142delG	p.D48fs	P	P	novel	M	15	15	4	1	No		PCC R-PGL	8.2	No
12	*SDHB*	E2	c.170A>C	p.H57P	LP	LP	novel	M	49	49		6	Yes	51	R- PGL	13	No
13	*SDHB*	E2	c.170delA	p.H57fs	P	P	novel	M	36	40	16	48	No		T- PGL	7	No
14	*SDHB*	E2	c.172dup	p.M58fs	P	P	novel	F	13	19	0	72	Yes	19	R- PGL	5	No
15	*SDHB*	E2	c.194T>G	p.L65R	P	P	23175444	M	32	32	1	1	No		R- PGL	8.1	No
16	*SDHB*	E3	c.229dup	p.I77fs	P	P	novel	M	53	60	0	84	Yes	60	R- PGL	7	No
17	*SDHB*	E3	c.277T>C	p.C93R	P	P	17848412	F	48	54	0	72	No		R- PGL	9	No
18	*SDHB*	E4	c.331_332del	p.L111fs	P	P	19454582	M	26	26	7	8	No		R- PGL	5.3	No
19	*SDHB*	E4	c.343C>G	p.R115G	P	P	rs751000085	M	61	62	7	6	No		PCC	3.3	No
20	*SDHB*	E4	c.386C>G	p.P129R	LP	LP	rs587781735	M	14	14	1	3	Yes	14	R- PGL	1.8	No
21	*SDHB*	E4	c.416T>G	p.L139R	P	P	27279923	F	42	47	1	60	No		R- PGL	4.2	No
22	*SDHB*	E6	c.552C>G	p.Y184X	P	P	29386252	F	9	10	2	12	Yes	12	R- PGL	6	No
23	*SDHB*	E6	c.574T>G	p.C192G	P	P	12000816	F	15	15	15	5	Yes	24	Multiple R- PGL	4.4	No
24	*SDHB*	E7	c.662A>G	p.D221G	LP	LP	novel	M	17	17	11	5	Yes	22	PCC	8	No
25	*SDHB*	E7	c.662A>G	p.D221G	LP	LP	novel	F	16	16	1	3	Yes	16	R- PGL		No
26	*SDHB*	E7	c.688C>T	p.R230C	P	P	22517557	M	51	56	0	60	No		R- PGL	8.7	Yes
27	*SDHB*	E7	c.688C>T	p.R230C	P	P	22517557	F	–	37		–	No		R- PGL		Yes
28	*SDHB*	E7	c.689G>A	p.R230H	P	P	16314641	F	18	28	5	120	No		R- PGL	6.5	No
29	*SDHB*	E7	c.689G>A	p.R230H	P	P	16314641	M	26	26	2	10	No		R- PGL	4.1	No
30	*SDHB*	E7	c.689G>A	p.R230H	P	P	16314641	F	17	19	1	24	No		R- PGL	4.8	No
31	*SDHB*	E7	c.725G>A	p.R242H	P	P	12000816	M	54	57	10	36	Yes	58	R- PGL	6.2	No
32	*SDHB*	E7	c.725G>A	p.R242H	P	P	12000816	M	59	59	1	5	Yes	60	R- PGL		No
33	*SDHB*	E7	c.725G>A	p.R242H	P	P	12000816	F	24	25	1	13	No		HN PGL	4.2	No
34	*SDHB*	E7	c.725G>A	p.R242H	P	P	12000816	M	10	10	0	1	Yes	13	R- PGL		No
35	*SDHB*		c.72+1G>T		P	P	16317055	M	39	45	1	72	No		R- PGL	5.1	No
36	*SDHB*		c.72+1G>T		P	P	16317055	M	13	13	3	6	Yes	16	R- PGL	8	Yes
37	*SDHB*		c.200+1G>C		P	P	19454582	M	10	10	4	1	No		PCC R-PGL	3.2	No
38	*SDHB*		c.423+1G>A		P	P	16405730	F	42	48	6	72	No		R- PGL	7.5	Yes
39	*SDHB*		c.423+1G>C		P	P	16314641	M	25	25	2	1	Yes	25	R- PGL	7.5	No
40	*SDHB*		c.765+1G>A		P	P	15328326	M	48	50	6	24	Yes	50	R- PGL	6.7	No
41	*SDHB*		c.765+1G>A		P	P	15328326	M	10	11	0	12	Yes	11	R- PGL	7	No
42	*SDHB*		c.765+1G>A		P	P	15328326	M	16	20	3	48	Yes	22	PCC	8.5	No
43	*SDHB*		c.766-1G>A		P	P	novel	M	6	16	4	120	Yes	19	R- PGL	5.3	Yes
44	*SDHB*		c.766-1G>A		P	P	novel	M	29	32	5	36	No		PCC	3.3	No
45	*SDHB*		c.766-1G>A		P	P	novel	F	27	27	2	1	No		R- PGL	14.4	No
46	*SDHB*	E1	Large deletions		P	P		M	25	28	2	36	No		R-PGL, HN-PGL	2.6	No
47	*SDHA*	E3	c.163T>C	p.Y55H	VUS^+^	VUS		M	58	62	2	48	No		R-PGL	5.0	No
48	*SDHA*	E3	c.163T>C	p.Y55H	VUS^*^	VUS		F	64	66	2	24	No		HN-PGL	1.9	No
49	*SDHA*	E4	c.323A>G	p.N108S	VUS^*^	VUS	rs758086385	M	61	61	12	3	No		PCC	7	No
50	*SDHA*	E5	c.460G>T	p.E154X	P	P	novel	F	61	61	1	1	No		R- PGL	4.4	No
51	*SDHA*	E5	c.508C>A	p.Q170K	VUS^−^	P	novel	F	30	40	2	120	No		P- PGL	2.9	No
52	*SDHA*	E5	c.562C>T	p.R188W	P	P	23282968	M	36	46	5	120	No		P- PGL	3	No
53	*SDHA*	E7	c.773G>T	p.G258V	VUS^−^	P	novel	F	12	13	7	6	No		R- PGL	12	No
54	*SDHA*	E8	c.935G>A	p.R312H	VUS^*^	VUS	novel	F	53	63	1	120	Yes	64	R-PGL	9.8	No
55	*SDHA*	E8	c.1054C>T	p.R352X	P	P	novel	M	61	63	1	24	No		R- PGL	4.5	No
56	*SDHA*	E8	c.1058A>G	p.E353G	VUS^*^	VUS	novel	M	39	41	5	18	No		R-PGL HN-PGL	7.2	No
57	*SDHA*	E9	c.1135C>T	p.R379C	VUS^+^	VUS	rs749309213	F	55	55	0	3	No		PCC	4.6	No
58	*SDHA*	E10	c.1334C>T	p.S445L	P	P	28384794	M	58	58	1	1	Yes	58	PCC	9	No
59	*SDHA*	E14	c.1865G>A	p.W622X	VUS^−^	P	23633203	F	21	33	1	144	No		R- PGL	4	No
60	*RET*	E10	c.1832G>A	p.C611Y	P	P	8557249	F	19	19	6	8	No		bil-PCC	9	No
61	*RET*	E11	c.1891G>T	p.D631Y	P	P	11149622	F	19	24	3	60	No		PCC	7	No
62	*RET*	E11	c.1892A>G	p.D631G	LP	LP	rs121913308	F		32	2		No		P- PGL		No
63	*RET*	E11	c.1900T>A	p.C634S	P	P	8099202	M	39	39	0	1	No		bil-PCC	4.1	Yes
64	*RET*	E11	c.1900T>C	p.C634R	P	P	8103403	F	30	33	3	36	No		bil-PCC	4.5	No
65	*RET*	E11	c.1900T>C	p.C634R	P	P	8103403	F	40	42	4	18	No		bil-PCC	5	No
66	*RET*	E11	c.1900T>C	p.C634R	P	P	8103403	F	31	31	1	2	No		PCC	4.6	Yes
67	*RET*	E11	c.1900T>G	p.C634G	P	P	8099202	F	21	21	4	7	Yes	25	bil-PCC	8	Yes
68	*RET*	E11	c.1900T>G	p.C634G	P	P	8099202	F	20	22	3	24	No		bil-PCC	4	Yes
69	*RET*	E11	c.1901G>A	p.C634Y	P	P	8099202	M	24	25	1	12	No		PCC	6	No
70	*RET*	E11	c.1901G>A	p.C634Y	P	P	8099202	F	39	39	16	6	No		bil-PCC	7.9	Yes
71	*RET*	E14	c.2410G>T	p.V804L	P	P	14718397	M	38	38	1	2	No		PCC	6.4	No
72	*SDHD*	E1	c.19C>G	p.L7V	VUS^+^	VUS	novel	F	29	59	0	360	No		R-PGL	9.6	No
73	*SDHD*	E1	c.24T>G	p.S8R	VUS^−^	LP	rs11550094	F	50	50	1	3	No		R- PGL	6.2	No
74	*SDHD*	E1	c.49C>T	p.R17X	P	P	18213727	M	26	26	2	2	Yes	28	HN PGL	4.6	No
75	*SDHD*	E2	c.64C>T	p.R22X	P	P	11391798	M	25	26	0	12	Yes	26	HN PGL	4.7	No
76	*SDHD*	E3	c.177_181del	p.S59fs	P	P	novel	M	23	23	32		No		PCC,R- PGL,HN PGL,T- PGL,	3.6	Yes
77	*SDHD*	E3	c.188_198del	p.S63fs	P	P	30484866	F	53	57	1	48	No		Multiple-HN PGL	3	No
78	*SDHD*	E3	c.217A>G	p.S73G	VUS^+^	VUS	rs748545223	F	43	43	1	2	Yes	52	R-PGL	5	No
79	*SDHD*	E3	c.217A>G	p.S73G	VUS^*^	VUS	rs748545223	M	23	29	0	72	No		PCC	6.5	No
80	*SDHD*	E3	c.305A>C	p.H102P	P	P	19454582	F		48	7		Yes	53	HN PGL		No
81	*SDHD*	E4	c.338A>T	p.D113V	LP	LP	rs786202513	F	24	26	6	24	No		R- PGL	5.3	Yes
82	*SDHD*		c.315-1G>T		P	P	novel	F	25	28	4	36	No		bil-PCC,R- PGL,HN PGL		No
83	*VHL*	E1	c.250G>A	p.V84M	P	P	17688370	F	24	30	0	72	No		R- PGL	4.1	No
84	*VHL*	E1	c.260T>A	p.V87E	P	P	28388566	M		12			No		bil-PCC		No
85	*VHL*	E1	c.314C>T	p.T105M	LP	LP	rs761240835	M	9	13	10	48	No		bil-PCC, R- PGL		No
86	*VHL*	E2	c.414A>G	p.P138P	P	P	30946460	M	15	15	2	3	No		bil-PCC	6.8	Yes
87	*VHL*	E2	c.414A>G	p.P138P	P	P	30946460	M	36	36	2	2	No		PCC	2.3	No
88	*VHL*	E2	c.458T>A	p.L153Q	P	P	24466223	M	13	22	0	156	No		bil-PCC	8	Yes
89	*VHL*	E2	c.460C>T	p.P154S	P	P	16595991	F		39	0		Yes	39	R- PGL	6.8	No
90	*VHL*	E3	c.482G>A	p.R161Q	P	P	7728151	M	19	19	4	6	No		bil-PCC, R- PGL	6.6	Yes
91	*VHL*	E3	c.482G>A	p.R161Q	P	P	7728151	F	10	10	18	6	Yes	28	bil-PCC, R- PGL	7	No
92	*VHL*	E3	c.499C>T	p.R167W	P	P	7987306	M	9	9	22		No		bil-PCC, R- PGL	5	No
93	*VHL*	E3	c.500G>A	p.R167Q	P	P	7987306	M	10	13	9	36	No		PCC	6.5	Yes
94	*VHL*	E3	c.500G>A	p.R167Q	P	P	7987306	M	14	14	31		No		PCC		No
95	*FH*	E2	c.193G>A	p.D65N	VUS^*^	VUS	rs769956664	M	57	57	5	6	No		PCC R-PGL	6.1	No
96	*FH*	E2	c.206G>A	p.G69D	VUS^*^	VUS	novel	F	26	27	6	12	No		PCC	4.5	No
97	*FH*	E6	c.799C>G	p.P267A	VUS^*^	VUS	novel	F	46	48	2	24	No		R-PGL	3.5	No
98	*FH*	E6	c.817G>A	p.A273T	P	P	&	F	30	36	2	72	No		P- PGL	2.5	Yes
99	*FH*	E9	c.1373C>T	p.A458V	VUS^*^	VUS	novel	M	46	61	0	180	No		PCC	6.3	No
100	*FH*	E10	c.1516A>G	p.M506V	VUS^*^	VUS	rs762413315	F	49	56	1	84	Yes	56	P-PGL		No
101	*MAX*	E1	c.1A>G	p.M1V	P	P	21685915	M	36	37	3	12	No		bil-PCC	5.9	No
102	*MAX*	E3	c.97C>T	p.R33X	P	P	21685915	M	16	18	8	24	No		PCC	4	Yes
103	*MAX*	E3	c.97C>T	p.R33X	P	P	21685915	F	31	31	17	3	Yes	48	PCC	10	No
104	*MAX*	E3	c.97C>T	p.R33X	P	P	21685915	F	39	43	2	48	No		bil-PCC	3.5	No
105	*MAX*	E3	c.97C>T	p.R33X	P	P	21685915	F	18	18	14		Yes	22	PCC	7	No
106	*MAX*	E4	c.223C>T	p.R75X	P	P	21685915	M	24	25	14	12	No		bil-PCC	3.5	No
107	*TMEM127*	E2	c.133T>C	p.C45R	VUS^*^	VUS	novel	M	27	27	2	3	No		PCC	5	No
108	*KIF1B*	E33	c.3649C>T	p.P1217S	P	P	18334619	F		36	0		Yes	36	PCC		No

&https://doi.org/10.1101/663609.

P represents for Pathogenic; LP represents for Likely Pathogenic; VUS represents for Variant of Uncertain Significance; F represents for female; M represents for male.

Age at dia. represents for Age at diagnosis; Age at meta. represents for Age at metastasis; Delay represents for delay between the PPGL diagnosis and the PPGL genetic testing; R represents for Retroperitoneal; P represents for Pelvic; T represents for Thoracic; HN represents for Head and neck; bil-PCC represents for bilateral pheochromocytoma.

^−^represents for SDHB immunohistochemical negative.

^+^represents for SDHB immunohistochemical positive.

^*^represents for FFPE unavailable.

**Table 2:** Clinical characteristics of different pathogenic genes in PPGL patients.

	SDHB (n = 46)	SDHD (n = 8)	SDHA (n = 7)	RET (n = 12)	VHL (n = 12)	MAX (n = 6)	FH (n = 1)	KIF1B (n = 1)
Sex(M/F)	27/19	3/5	3/4	3/9	9/3	3/3	0/1	0/1
Age (year)	26 (16, 41)	27 (26, 49)	46 (37, 60)	32 (23, 28)	15 (13, 24)	28 (20, 36)	36	36
Duration (month)	12 (5, 48)	18 (5, 33)	24 (4, 120)	8 (6, 21)	7 (5, 45)	12 (12, 24)	72	3
Delay (year)	2 (1, 6)	3 (1, 6)	1 (1, 4)	3 (2, 4)	4 (1, 14)	11 (4, 14)	2	0
Multiple	5/46 (10.9%)	3/8 (37.5%)	0	7/12 (58.3%)	7/12 (58.3%)	3/6 (50.0%)	0	0
Metastasis	25/46 (54.3%)	3/8 (37.5%)	1/7 (14.3%)	1/12 (8.3%)	2/12 (16.7%)	2/6 (33.3%)	0	1
Location								
PCC	6/46 (13.0%)	0	1/7 (14.3%)	4/12 (33.3%)	3/12 (25.0%)	3/6 (50.0%)	0	1
Bil-PCC	0	1/8(12.5%)	0	7/12 (58.3%)	7/12 (58.3%)	3/6 (50.0%)	0	0
PGL	41/46 (89.1%)	8/8(100.0%)	6/7 (85.7%)	1/12 (8.3%)	3/12 (25.0%)		1	0
HN-PGL	2/46 (4.3%)	6/8(75.0%)	0	0	0	0	0	0
T-PGL	1/46 (2.1%)	1/8(12.5%)	0	0	0	0	0	0
R-PGL	38/46 (82.6%)	4/8(50.0%)	4/7 (57.1%)	0	3/12 (25.0%)	0	0	0
P-PGL	2/46 (4.3%)	0	2/7 (28.6%)	1/12 (8.3%)	0	0	1	0
PCC+PGL	1/46 (2.1%)	2/8(25.0%)	0		2/12 (16.7%)	0	0	0
Family History	5/46 (10.9%)	2/8(25.0%)	0	5/12 (41.7%)	0	1/6(16.7%)	1	0
Catecholamine								
NE	271.3 (126.0, 967.5)	148.0 (81.7, 211.0)	135.0 (51.9, 246.7)	77.6 (38.6, 364.8)	502.6 (300.7, 786.4)	343.4 (303.1, 399.8)	31.4	256.9
E	3.5 (2.6, 4.6)	3.0 (2, 3.7.0)	4.0 (3, 4.3)	6.1 (3.4, 36.7)	3.6 (2.9, 4.7)	4.7 (4.0, 7.3)	4.4	105.8
DA	274.5 (220.9, 462.1)	171 (127.1, 212.5)	258.4 (176, 295.4)	287.7 (187.6, 326.8)	253.3 (217.3, 302.6)	389.8 (211, 617.1)	326	284.2
Tumor size(cm)	6.4(4.5, 8.0)	4.7 (3.9, 5.2)	4.4 (3.5, 6.8)	6.0 (4.5, 7.5)	6.6 (5.0, 6.8)	5.0 (3.6, 6.7)	2.5	NA

Data are medians and interquartile ranges.

PCC represents for pheochromocytoma; PGL represents for paraganglioma; R represents for Retroperitoneal; P represents for Pelvic; T represents for Thoracic; HN represents for Head and neck; bil-PCC represents for bilateral pheochromocytoma.

NE represents for 24-h urinary Norepinephrine (normal range:16.7~40.7 μg/24 h); E represents for 24-h urinary Epinephrine (normal range: 1.7~6.4 μg/24 h); DA represents for 24-h urinary Dopamine (normal range: 120.9~330.6 μg/24 h).

NA represents for unavailable.

### The Profile of Gene Variants

**Figure 2:**
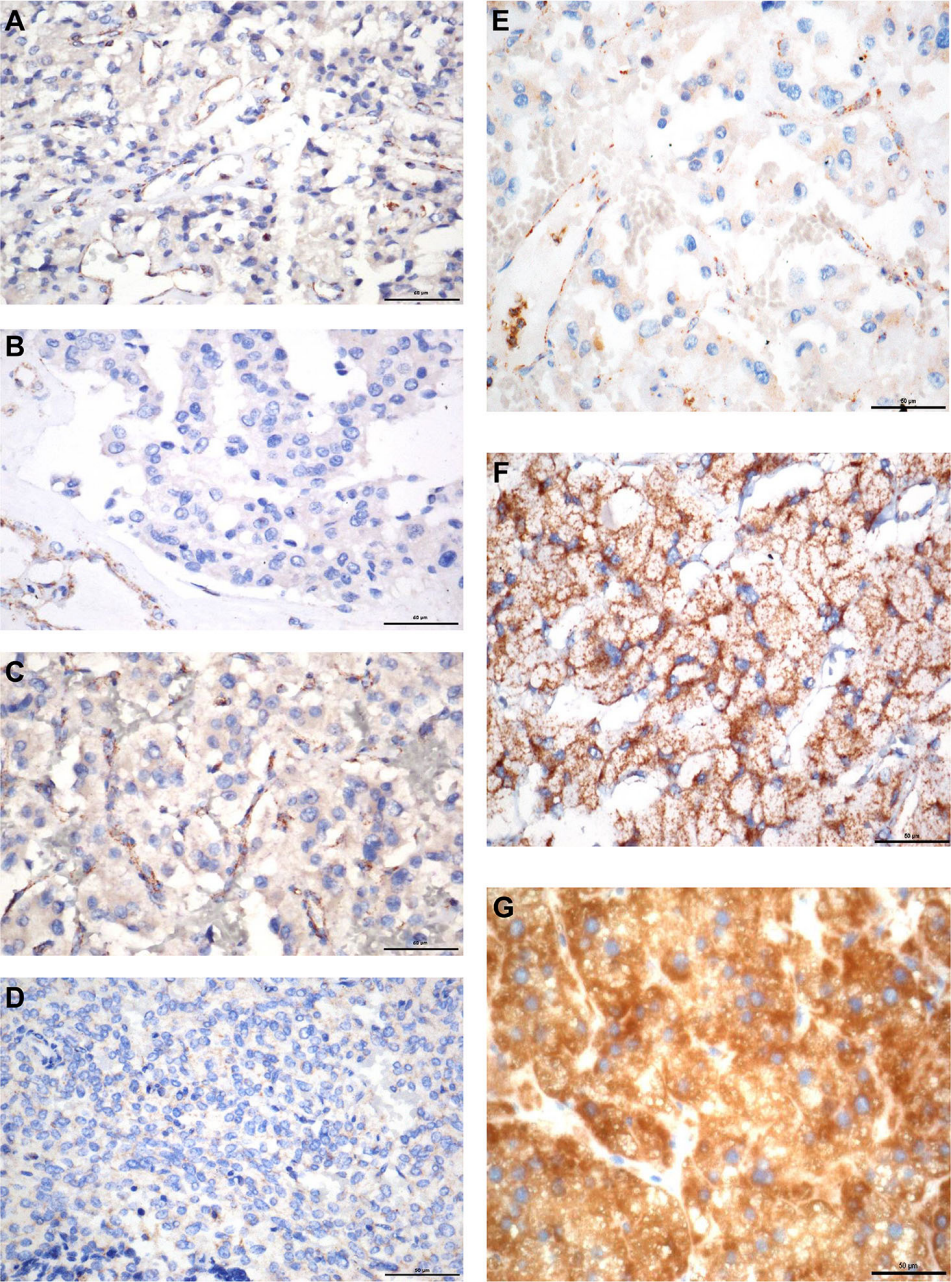
SDHB immunohistochemistry. **(A)** PPGL with *SDHA* mutation (c.C508A). **(B)** PPGL with *SDHA* mutation (c.G773T). **(C)** PPGL with *SDHA* mutation (c.G1865A). **(D)** PPGL with *SDHD* mutation (c.T24G). **(E)** PPGL with an exon 1 large deletion of *SDHB*. **(F)** PPGL with *RET* mutation. **(G)** Normal adrenal medulla. Note: Absence of SDHB immunostaining in the tumour cells **(A**–**E)**, with positive staining in the normal cells of the intratumoral fibrovascular network. Strong granular staining in PPGL with RET mutation and normal adrenal medulla **(F**, **G)**.

We found 107 variants in 314 cases (34.1%) (**Table 1**). Among them, 76/107 variants (65.4%) were previously reported as pathogenic or likely pathogenic, 8/107 variants were uncertainly significant, and 23/107 variants (21.5%) were novel. According to the guidelines of ACMG, 13/23 (56.5%) novel variations were classified as pathogenic or likely pathogenic, 10 variants remained of uncertain significance. SDHB immunohistochemistry was performed on available FFEP tissue sections with VUS of *SDHx*. Negative immunostaining of SDHB was presented in 3 VUS of *SDHA* and one VUS of *SDHD* (**Figure 2**). Positive immunostaining of SDHB was presented in 2 VUS of *SDHA* and 2 VUS of *SDHD*, which suggests variants may not be pathogenic (**Supplementary Figure S1**).

In 207 cases without variants in 12 common pathogenic genes. SDHB immunohistochemistry was performed in 96/207 FFPE tissue sections, only one sample presented negative immunostaining of SDHB. MLPA analysis of the *SDHx* was then used in the immunohistochemistry negative sample and another 43 samples which contain multiple, metastatic PPGL. MLPA analysis of the *VHL* was used in early onset (under 20 years old) (n = 19), bilateral PCC (n = 2) or PCC and PGL (n = 6) in succession. We did not detect any deletions and duplications of *VHL*. Deletions of *SDHB* gene were detected in the negative immunostaining sample, affecting exon 1.

Altogether, 93/314(29.6%) of PPGLs had germline mutations in 12 known pathogenic genes.*SDHB* was the most frequently mutated gene (46/314, 14.6%), followed by *RET* (12/314, 3.8%), *VHL* (12/314, 3.8%), *SDHD* (8/314, 2.5%), *SDHA* (7/314, 2.2%), *MAX* (6/314, 1.9%), *FH* (1/314, 0.3%), and *KIF1B* (1/314, 0.3%).

#### *SDHB* Variants

*SDHB* was the most commonly mutated gene in our series (46/93 variants, 49.5%) with a median age at diagnosis of 26. Compared to sporadic PPGL, PPGL with *SDHB* variants were more likely to develop distant metastases or PGL (**Table 3**). 25/46(54.3%) of cases developed metastasis with the median duration of 12 (5, 51) months. Eighty-five percent (39/46) of the cases were PGL, 5/46 of the cases were PCC and 2/46 of the cases had both PCC and PGL (**Table 2**). In our cohort, *SDHB* variants were mainly located in exon 2 and exon 7, which accounted for 26/46 (56.5%) of all *SDHB* variants. Among them, codon 46 in exon 2 and codons 230 and 242 in exon 7 were hot variant sites, and were found in 8, 5, and 4 patients, respectively. Besides, variants in IVS7 (c.765+1G>A and c.766−1G>A) were also common in our study and the rate was 6/46 (13.0%).Four unrelated individuals had a family history of PPGL. Two PPGL complicated with pancreatic neuroendocrine tumor and renal clear cell carcinoma, respectively.

**Table 3:** Genotype-phenotype correlation in patients with PPGL.

	Metastasis		Location			Age(years)		Family History	
	Yes n = (69)	No n = (245)	PCC n = (141)	PGL n = (156)	Multiple (PCC and PGL) n = (17)	<30 n = (105)	>30 n = (209)	Yes n = (18)	No n = (296)
SDHB (n = 46)	25/69 (36.2%)	21/245 (8.6%)	5/141 (3.5%)	39/156 (25.0%)	2/17 (11.8%)	28/105(26.7%)^*^	18/209 (8.6%)	5/18 (27.8%)	40/296 (13.5%)
SDHD (n = 8)	3/69 (4.3%)	5/245 (2.0%)	0	6/156 (3.8%)	2/17 (11.8%)	5/105 (4.8%)	2/209 (1.0%)	2/18 (11.1%)	6/296 (2.0%)
SDHA (n = 7)	1/69 (1.4%)	6/245 (2.4%)	1/141 (0.7%)	6/156 (3.8%)	0	1/105 (1.0%)	6/209 (2.9%)	0	7/296 (2.4%)
RET (n = 12)	1/69 (1.4%)	11/245 (4.5%)	11/141 (7.8%)	1/156 (0.6%)	0	5/105 (4.8%)	7/209 (3.3%)	5/18 (27.8%)	7/296 (2.4%)
VHL (n = 12)	2/69 (2.9%)	10/245 (4.1%)	6/141 (5.0%)	3/156 (1.9%)	2/17 (11.8%)	10/105 (9.5%)^*^	2/209 (1.0%)	4/18 (22.2%)	8/296 (2.7%)
MAX (n = 6)	2/69 (2.9%)	4/245 (1.6%)	6/141 (4.3%)	0	0	3/105 (2.9%)	6/209 (2.9%)	1/18 (5.6%)	5/296 (1.7%)
FH (n = 1)	0	1/245 (0.4%)	0	1/156 (0.6%)	0	0	1/209 (0.5%)	1/18 (5.6%)	0
KIF1B (n = 1)	1/69 (1.4%)	0	1/141 (0.7%)	0	0	0	1/209 (0.5%)	0	1/296 (0.3%)
12 pathogenic genes(n = 93)	35/69 (50.7%)^*^	58/245 (23.7%)	30/141 (21.3%)	56/156 (35.9%)^#^	6/17 (35.3%)	52/105 (49.5%)^*^	43/209 (20.6%)	18^*^	75/296 (25.3%)
Sporadic(n = 206)	31/69 (44.9%)	175/245 (71.4%)	105/141 (74.5%)	93/156 (59.6%)	8/17 (47.1%)	50/105 (47.6%)	156/209 (74.6%)	0	206/296 (69.6%)

^*^P < 0.001 vs. sporadic group.

^#^P < 0.05 vs. sporadic group.

**Figure 3:**
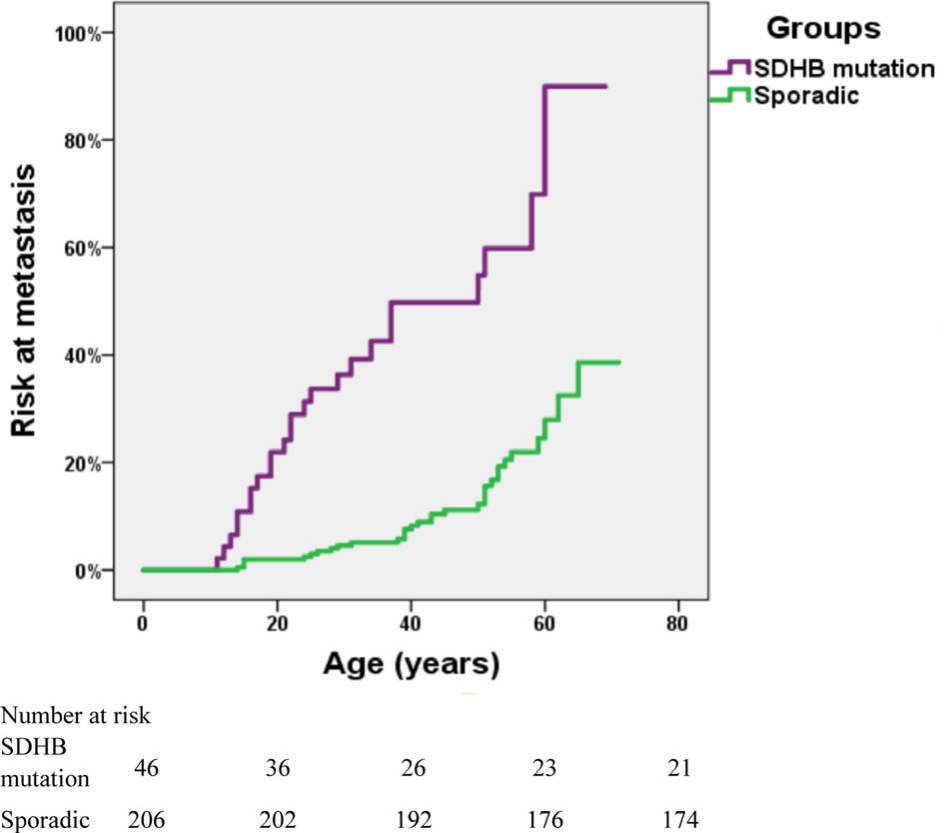
Risk of metastasis in *SDHB*-mutated and sporadic PPGL patients. Kaplan-Meier analysis includes 46 *SDHB* (succinate dehydrogenase subunit B) mutated PPGLs and 206 apparently sporadic PPGLs.

The risk of metastasis in *SDHB*-mutated and sporadic PPGL patients was shown in **Figure 3**. There was a statistically significant difference in the risk of development into distant metastasis between patients with *SDHB* variants and sporadic PPGLs (P <.001). For PPGLs with *SDHB* variants, the risk of metastasis by the age of 60 was close to 60%, while in patients with sporadic PPGL, the risk was roughly 20% (P <.001) (**Figure 3**).

#### *SDHA* Variants

There were 13 cases with SDHA variants. Seven patients with pathogenic SDHA variants were diagnosed at a median age of 46 (range: from 13 to 63 years). Most of the patients had PGL (6/7, 85.7%), and of them, 4 had retroperitoneal PGL and 2 had pelvic PGL. One cases developed metastasis (**Table 2**). Exon 5 tended to have recurrent variants. There was no family history or syndrome in PPGLs with SDHA variants.

#### *SDHD* Variants

There were 11 cases with *SDHD* variants while 8 variants were verified as pathogenic and 3 remained VUS. Cases carrying pathogenic *SDHD* mutation were diagnosed at a median age of 27 (range: from 23 to 57 years), and 99mTc-HYNIC-TOC scintigraphy was used for the detection of head and neck PGL or multiple PGL in *SDHD* mutation carriers. All cases had PGL. PGL was in head and neck in 4 and in retroperitoneal cavity in 2. Thirty seven percent (3/8) of cases had multiple PPGL. The mutation type of 3 cases with multiple PPGL were framshifts or splicing. Three cases with single nucleotide variants, mostly (2/3, 66.7%) nonsence mutation, tend to develop into single head and neck PGL and metastasis. Two patients had both PCC and PGL.The variant scattered across the 4 exons of *SDHD*. Two cases had a family history of PPGL, and one case had pituitary growth hormone adenoma in addition to PPGL.

#### *RET* Variants

*RET* variants were found in 12 patients. Ninety one percent (11/12) of patients had PCC, among which, 7 had bilateral PCC. Variants in codon 631 and codon 634 in exon 11 of *RET*, were found in 10/12 (83.3%) cases. Forty one percent (5/12) had PPGL family history. In addition to PPGL, 6 patients also had clinical manifestations of medullary thyroid carcinoma. One patient had both PCC and primary hyperparathyroidism.

#### *VHL* Variants

*VHL* variants were detected in 12 patients. Six cases had PCC, including 3 bilateral PCC. Two patients had retroperitoneal PGL, and 4 patients had both bilateral PCC and retroperitoneal PGL (**Table 1**). Two metastatic PPGL which had no proof of renal cell carcinoma were also found in *VHL* mutated patients. Variants in codon 161 and 167, the most frequently reported mutant sites, were detected in 5/12(41.7%) patients. Four patients had family history of PPGL.And two cases had other clinical manifestations of VHL disease, such as bilateral retinoblastoma, cerebellar hemangioblastoma, renal clear cell carcinoma, and pancreatic neuroendocrine tumor in addition to PPGL.

Compared to sporadic PPGLs, pathogenic germline variants, especially *SDHB* and *VHL*, were more likely to be detected in patients under 30 (P <.001).

#### Other Gene Variants

Six cases had *MAX* variants, and all of them were PCC and half of the PCC were bilateral. Five variants were nonsense and one disrupted the *MAX* protein by affecting the initial methionine (c.1A>G). The most frequent variant was c.97C>T, p.Arg33Ter (4/6, 66.7%), and 2 of the 4 patients with such variant had metastatic PPGL. One of the patients with *MAX* variants had family history. In addition to PCC, two patients had pituitary growth hormone/prolactin adenoma and thyroid papillary carcinoma, respectively. One case with a family history of PPGL carried a *FH* pathogenic variant. A patient with *KIF1B* variant had metastatic PCC.

### Comparison Between Apparently Sporadic PPGLs and Their Counterparts

In our cohort, further analysis was conducted in 206 patients with apparently sporadic PPGL (i.e., patients without variants in 12 known pathogenic genes) and 25 cases with family history or syndromic presentation (**Table 4**). Compared to apparently sporadic PPGLs, patients with family history or syndromic presentation had an earlier age of onset (P <.000), a higher level of norepinephrine (P <.038) and were more likely to develop multiple PPGL (P <.000). Besides, PCC was more common in apparently sporadic PPGLs (P = 0.014).

**Table 4:** Comparison of the clinical characteristics between PPGLs with family history or syndromic presentation and apparently sporadic PPGLs.

	PPGLs with family historyor syndromic presentation (n = 25)	PPGLs withapparently sporadic presentation(n = 206)	P-value
Age at onset (year)	24 (16, 38)	38 (30, 47)	0.000
Age at diagnosis (year)	25 (18, 37)	42 (31, 52)	0.000
Duration (month)	24 (8, 54)	24 (4, 60)	0.297
Metastasis	4/25 (16%)	32/206 (16%)	1.000
PCC	6/25 (24%)	103/206 (50%)	0.014
PGL	7/25 (28%)	89/206 (43%)	0.145
Multiple PPGL	12/25 (48%)	14/206 (7%)	0.000
Tumor size (cm)	6.0 (4.8, 8.0)	5.4 (4.0, 7.2)	0.260
NE	348.8 (141.0, 542.8)	113.7 (39.4, 440.3)	0.038
E	4.4 (2.9, 7.4)	4.0 (2.7, 6.7)	0.700
DA	285.1 (204.8, 350.0)	230.1 (172.2, 329.1)	0.300

Data are medians and interquartile ranges.

PCC represents for pheochromocytoma; PGL represents for paraganglioma; PPGL represents for pheochromocytoma and paraganglioma; NE represents for 24-h urinary Norepinephrine (normal range:16.7~40.7μg/24 h); E represents for 24-h urinary Epinephrine (normal range: 1.7~6.4 μg/24 h); DA represents for 24-h urinary Dopamine (normal range: 120.9~330.6 μg/24 h).

### Variants in PCC and PGL

There were 141 PCC in 314 PPGLs. The 141 PCC patients included 15 bilateral PCC and 21 metastatic PCC patients. Thirty PCC patients (30/141, 21.3%) had germline variants (**Figure 1** and **Table 3**). The top two most frequently mutated genes were *RET* (11/141, 7.8%) and *VHL* (6/141, 4.3%), followed by *MAX* (6/141, 4.3%), *SDHB* (5/141, 3.5%), *SDHA* (1/141, 0.7%), and *KIF1B* (1/141, 0.7%).

Twenty bilateral PCC were included in our cohort (**Figure 1**), 5 were complicated with PGL. 7/20 (35%) had *RET* variants, 7/20 (35%) had *VHL* variants, 3/20 (15%) had *MAX* variants, 1/20 (5%) had *SDHD* variant, and 2/20 (10%) had no known germline variants (**Table 2**).

One hundred and fifty six PGL patients were included in our research, involving 129 retroperitoneal PGL, 18 pelvic PGL, 9 head and neck PGL, and 4 thoracic PGL (**Table 2**). Thirty six percent (56/156) of the PGLs had known pathogenic gene variants (**Table 3**). In 129 retroperitoneal PGL (**Table 2**), 34.1% of them had germline mutations. The most frequently mutated gene was *SDHB* (36/129, 27.9%), followed by *SDHA* (4/129, 3.1%), *VHL* (2/129, 1.6%), *SDHD* (1/129, 0.8%), and *RET* (1/129, 0.8%). In 18 pelvic PGL patients, 33.3% of them had germline variants, including *SDHB* (2/18, 11.1%), *SDHA* (2/18, 11.1%), *RET* (1/18, 5.6%), and *FH* (1/18, 5.6%). In 9 patients with head and neck PGL (**Figure 1**), 66.7% of the patients had germline variants, including *SDHD* (4/9, 44.4%) and *SDHB* (2/9, 22.2%). Thoracic PGL were related to *SDHB* (1/4, 25%) and sporadic PPGL (3/4, 75%). Five patients had both bilateral PCC and PGL. All of them had pathogenic variants, of which 4 carried *VHL* mutation and 1 carried *SDHD* mutation.

Compared to sporadic PPGLs, PPGLs with variants in known pathogenic genes were more likely to develop PGL (P <.001).

### Genetic Alterations in Metastatic PPGLs

Pathogenic germline variants were more likely to occur in metastatic PPGL than in their non-metastatic counterparts [35/69 (50.7%) vs. 58/245 (23.7%), P <.001]. Moreover, distant metastasis occurred more often in PGL than in PCC [45/156 (28.8%) in PGL vs. 21/141 (14.9%) in PCC, P = 0.004]. In metastatic PPGL, *SDHB* (25/69, 36.2%) was the most commonly mutated gene (**Figure 1** and **Table 3**). *SDHD* (3/69, 4.3%), *MAX* (2/69, 2.9%), and *VHL* (2/69, 2.9%) had relatively higher frequency of variants. *SDHA*, *RET*, and *KIF1B* were also found to have variants, with a rate of 1.4% (1/69), while we did not found mutations in *FH* in metastatic PPGLs. Of note, 31/69 (44.9%) metastatic PPGL did not possess 12 known pathogenic genes (**Figure 1** and **Table 3**).

### Catecholamine Phenotype in PPGLs

Twenty four–hour urinary catecholamine excretion charactered elevated NE or elevated NE and DA in *SDHB* mutated PPGLs. Most patients carrying mutations in *SDHD*, *SDHA*, or *MAX* had elevated NE.

## Discussion

In this cohort of Chinese PPGL patients, we profiled the genetic variants and demonstrated genotype-phenotype correlation in Chinese PPGL patients. We found that germline variant rate in PPGL patients was up to 29.6%, and *SDHB* was the most frequently mutated gene. The genetic and clinical features of Chinese PPGL patients were unique in some aspects compared to their non-Chinese counterparts.

The overall variant rates of pathogenic genes in PPGLs in our cohort were similar to those reported previously, standing somewhere between 11% to over 40% (7, 20, 21). A study conducted in Italy examined 10 genes in 501 PPGL patients and found that germline variant rate was 32.1%, and *VHL* was the most frequently mutated gene (22). A recent study from Saudi Arabia reveals that the variant rate was 36.6%, and *SDHB* was the most common variant (9). Jiang J et al. recently reported 719 PPGLs from two centers in China, of the 719 PPGLs, 266 cases were included in the analysis of germline mutations. Almost twenty one percent of PPGL had pathogenic germline mutations and *VHL* was mostly mutated in PPGL at a rate of 23/261(8.8%) in germline level (10). Compared to the research of Jiang J et al., our single center had a higher mutation rate of 29.6% and *SDHB* was the most commonly mutated gene in our center. The difference may come from that patients were referred to our hospital from all over the country mostly for their relative complicated or hard-treated diseases. Moreover, the proportion of metastatic PPGL and extra-adrenal PGL in a cohort might also affect the profile of genetic variants.

It is noteworthy that the variant hotspots of *SDHB* varied with different cohorts. According to TCGA data from 173 PPGL patients (23), the detection rate of *SDHB* germline variant was 9.8% (17/173). Among the 17 *SDHB* mutated PPGLs, p.Arg46Ter variant, which was the most frequently mutated one in our *SDHB* mutated PPGLs (6/45, 13.3%), occurred in only one patient (1/17, 5.9%). And the most frequent *SDHB* variant in TCGA data was p.Ile127Ser [29.4% (5/17)], which was not detected in our cohort. In a research conducted in Saudi Arabia, the most common *SDHB* variant was p.Arg90Ter, occurring in 57% of all the *SDHB* mutated cases, while in our series, no case had such variant (9). A national study from the Netherlands revealed that IVS4 (c.423+1G>A) was the most common variant site and was detected in 16/83 (19%) *SDHB* mutated patients, while the variant was found in 2/45 (4.4%) *SDHB* mutated patient in our cohort (24). In addition, in our study, novel variants c.765-1G>A and p.Asp221Gly in *SDHB* were recurrent. Exon 2, exon 7, and the splicing of IVS7 (c.765+1G>A and c.766-1G>A) were the most commonly mutated domains in our cohorts, and the finding was different from the results of other large sample researches (24–27). All the aforementioned studies, including ours, strongly suggest that the hotspots of *SDHB* variant vary in different races or countries, and it may be feasible to preferentially detect some specific sites of *SDHB* gene for metastatic PPGL patients according to the variant hotspots to achieve better cost-effectiveness and efficiency. The clinical features of patients with *SDHB* variants in our series mimicked those in other studies, and *SDHB* variant is associated with development of metastasis and extra-adrenal tumors.

Our results revealed that patients under 30 had a high germline variant rate (52.6%), suggesting that it is necessary to screen the genes in young patients. On the other hand, with *SDHA* and *SDHD* variants, corresponding tumors tend to affect elderly patients (23). It has been reported that the *SDHD* variant carrier had a penetrance rate of 86% at the age of 50 (22, 24, 25). In our study, only 2/8 (25%) *SDHD*-mutated patients had family history of PPGL. Family history may be underestimated because it was obtained by enquiring rather than systematic examination and gene detection in family members.

In our cohort, the hotspot variants of *RET* and *VHL* genes were similar to those reported previously, and were at codons 631 and 634 in exon 11 of RET gene and at codons 161 and 167 in exon 3 of *VHL* gene (26, 27). Of note, a synonymous variant recently reported in *VHL* was found in two patients in our study (28). *RET* and *VHL* gene variants mainly cause PCC, and they are principally responsible for development of bilateral PCC, accounting for 70% in all bilateral PCC in our study. Previous studies also found PGL in *RET*- and *VHL*-mutated patients (26, 27). In our series, only one patient (1/12, 8.3%) with *RET* variant developed PGL. But in *VHL*-mutated patients, 50% (6/12 cases) had retroperitoneal PGL. The rate was higher than that of previous research (10%–20%) (26). Our study suggested that although *VHL* and *RET* gene variants mainly lead to adrenal PCC, they may also result in extra-adrenal PGL. This is especially true of *VHL* variant, which needs to be screened carefully in clinical practice.

Up to now, only 12 PPGL patients with *FH* gene variants have been reported (29–31). In our cohort, we identified six *FH* variants in 314 individuals with PPGL. Only one of the *FH* variants was identified to be pathogenic variants against ACMG. The first published report showed that metastatic PPGLs accounted for 60% (3/5 cases) in *FH*-mutated PPGLs. Nonetheless, no evidence of metastasis of the PPGL with *FH* mutation in our cohort has been found till now.

*MAX* has been recently described at a total of ~40 PPGLs (9, 32–34). In our study, 6 patients had *MAX* variant, with the ratio being 1.9% in all PPGL patients. All *MAX*-mutated patients developed PCC in our cohort, which was coincident with previous studies (35). The frequent variant c.97C>T was also common in European and American populations (8/23 vs. 4/6 in our study) (35). Interestingly, of all the 4 PPGL cases carrying the same variant site c.97C>T, two were metastatic cases, while previous studies (26, 35) showed that patients carrying c.97C>T did not develop metastasis. This might be a unique genotype-phenotype correlation in Chinese.

*KIF1B* gene variant had been reported previously in a familial PCC patient. Both the proband and his grandfather had bilateral PCC without distant metastasis. Besides, the proband was also diagnosed with a neuroblastoma and a large well-differentiated leiomyosarcoma successively (36). Our research found a case of *KIF1B* variant who had a unilateral PCC with a metastatic lesion. Of note, the variant site had been previously reported in a patient with neuroblastoma (37). Our case suggested that the germline variants of the *KIF1B* gene could also pose risk of metastatic PCC.

Metastatic PPGL was reported to make up 2% to 13% (vs. 21/141, 14.9% in our study) in PCC and 2.4% to 50% (vs. 45/156, 28.8% in our study) in PGL (38). In our cohort, 50.7% (35/69) of metastatic PPGLs had hereditary germline variants, and were mostly caused by *SDHB* variant, followed by *SDHD*, *VHL*, *MAX*, *SDHA*, *RET*, and *KIF1B*. The results were in line with previous reports (26). The median age at diagnose in metastatic PPGLs was 7 years older (32 years vs. 39 years in our study) than the previously reported age (38, 39). PPGLs with *SDHB* variants need more attention since they are highly predisposed to distant metastasis.

Our study had several limitations. The research focused on the most common 12 pathogenic genes of PPGL and registered a variant detection rate of 29.6%. Our study did not include rare pathogenic genes such as DLST, SLC25A11 and DNMT3A, etc., which might lower the detection rate of pathogenic mutations. Furthermore, next-generation sequencing used in this study could not detect large deletions of some pathogenic genes, such as *SDHx* and *VHL*. Nevertheless, we added SDHB immunohistochemistry technique and MLPA detection to all samples with multiple PPGL or metastasis or early onset that are most likely to have large deletions. The proportion of large deletions in *SDHx* was 1.6% (1/61), lower than previously reported.

## Conclusions

In conclusion, our study profiled the genetic variants and identified the corresponding clinical characteristics of PPGL in Chinese subjects. *SDHB* mutation hotspots are different between Chinese and other races. The genotype-phenotype relationship in Chinese is helpful in the prioritization of genetic tests and clinical decision-making.

## Data Availability Statement

The raw sequence data reported in the manuscript have been deposited in the Genome Sequence Archive in National Genomics Data Center, Beijing Institute of Genomics (China National Center for Bioinformation), Chinese Academy of Sciences, under accession number HRA000415 that are accessible at https://bigd.big.ac.cn/gsa-human/s/sKCKPT0A.

## Ethics Statement

The studies involving human participants were reviewed and approved by Medical Ethics Committee at Peking Union Medical College Hospital and written informed consent to participate in the study was provided by participants or their legal guardians/next of kin. Written informed consent to participate in this study was provided by the participants’ legal guardian/next of kin.

## Author Contributions

XM conducted the experiments and drafted the manuscript. ML conducted the experiments and analyzed the data. FW, YC, SC, XZ, and YZ collected the specimens. YL and AT reviewed the manuscript. All authors contributed to the article and approved the submitted version.

## Funding

This research was funded by CAMS Innovation Fund for Medical Sciences (CIFMS), grant number 2017-I2M-1-001.

## Conflict of Interest

The authors declare that the research was conducted in the absence of any commercial or financial relationships that could be construed as a potential conflict of interest.

## Supplementary Material

The Supplementary Material for this article can be found online at: https://www.frontiersin.org/articles/10.3389/fendo.2020.574662/full#supplementary-material. The Supplementary Material (Table_1.xlsx) for this article can be found online at: https://doi.org/10.6084/m9.figshare.12034230.v1
