# Supplementary material for: Genetic and Clinical Profiles of Pheochromocytoma and Paraganglioma: A Single Center Study
Source: Front Endocrinol (Lausanne). 2020 Dec 11;11:574662. doi: 10.3389/fendo.2020.574662 (PMC7761866; doi:10.3389/fendo.2020.574662)
Supplement: Supplementary file 1 [file Image_1.pdf]

Supplementary figure S1 PPGL with positive SDHB immunohistochemistry.

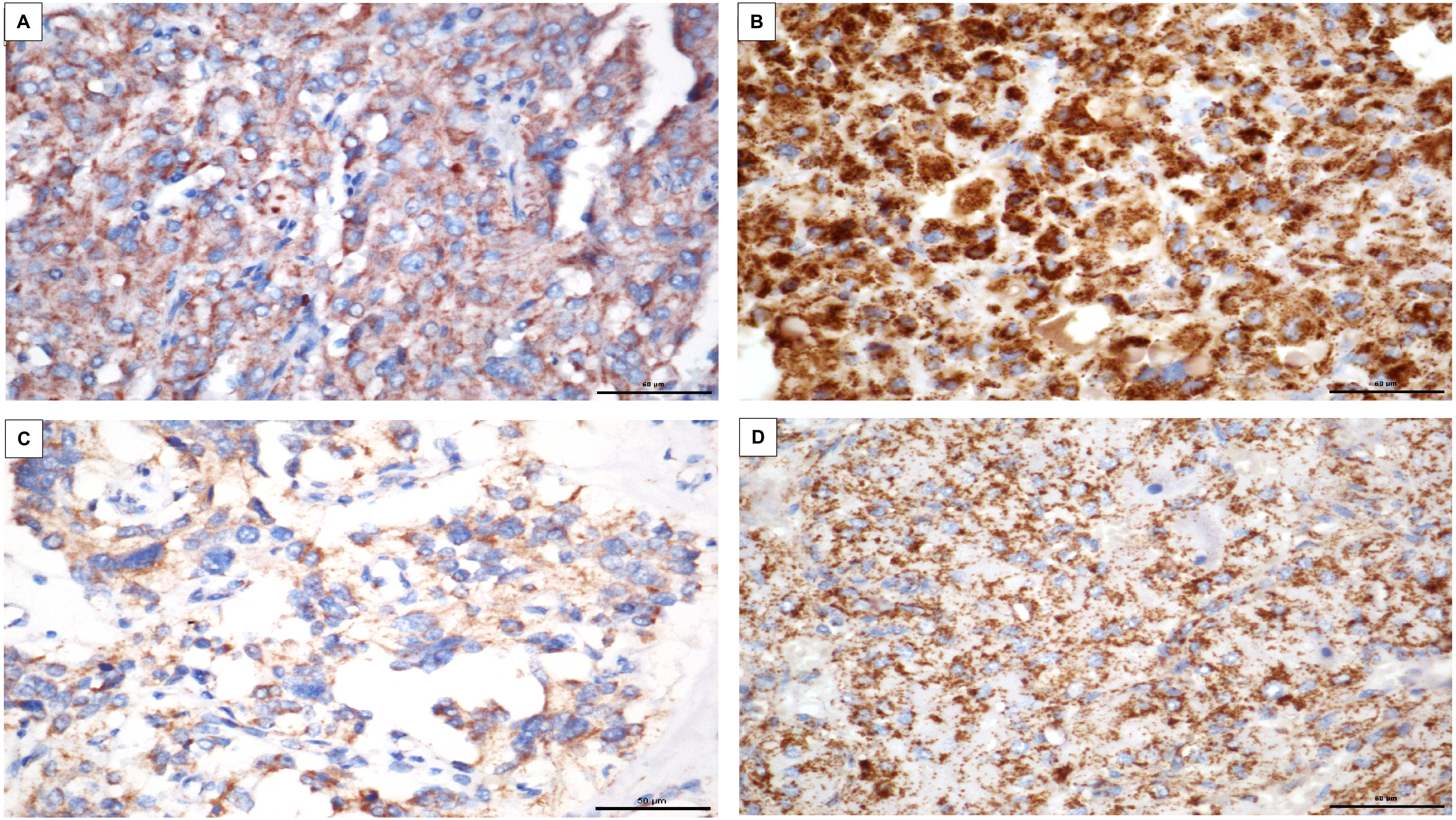

(A) PPGL with *SDHA* variant (c.T163C). (B) PPGL with *SDHA* variant (c.C1135T). (C) PPGL with *SDHD* variant (c.C19G). (D) PPGL with *SDHD* variant (c.A217G).

Note: Granular staining in the tumor cells.
